# THE CONTRIBUTION OF NIGROSTRIATAL DOPAMINERGIC TRANSMISSION ON APATHY IN OLDER ADULTS

**DOI:** 10.1093/geroni/igae098.3349

**Published:** 2024-12-31

**Authors:** Yixiao Song, Peggy Cawthon, Meryl Butters, Lana Chahine, Caterina Rosano

**Affiliations:** University of Pittsburgh, Pittsburgh, Pennsylvania, United States; California Pacific Medical Center, San Francisco, California, United States; University of Pittsburgh, Pittsburgh, Pennsylvania, United States; University of Pittsburgh, Pittsburgh, Pennsylvania, United States; University of Pittsburgh, Pittsburgh, Pennsylvania, United States

## Abstract

Apathy is a common and impairing symptom in neurological disorders and its connection with striatal dopamine levels has been examined in Parkinson’s and Alzheimer’s disease. Little is known about the nature of this relationship in older adults free from neurological diseases. This study explores the link between apathy and nigrostriatal dopaminergic neurotransmission in community-dwelling older adults without overt neurological symptoms. This was a cross-sectional study of community-dwelling older adults (N=230, mean age 75.3yrs; 60% female) from Monongahela-Youghiogheny Healthy Aging Team (MYHAT) and Study of Muscle, Mobility and Aging (SOMMA) studies. The nigrostriatal dopamine binding site density of VMAT2 was quantified via (+)-[11C]DTBZ PET imaging. Apathy was assessed with Apathy Evaluation Scale, an 18-item questionnaire using a 4-point Likert scale (range 18-72; higher score indicates greater apathy). Latent class analysis was used to identify latent subtypes of apathy. Multinomial logistic regression was used to investigate the relationship between dopaminergic transmission and latent classes. Three latent classes were identified, namely, low apathy (mean 20.7; SD 2.36; N=110), moderate apathy (mean 25.5; SD 2.83; N=89), and high apathy (mean 32; SD 4.82; N=31). Each SD decrement in nigrostriatal dopaminergic transmission corresponded to 39% and 42% lower odds of having high apathy compared to low apathy (OR=0.61; 95%CI: 0.39-0.95; p-value=0.028) and moderate apathy (OR=0.58; 95%CI: 0.36-0.90; p-value=0.015), respectively, after adjusting for age at the time of MRPET scan, gender, and race. Our study highlights apathy’s inverse relationship with dopaminergic neurotransmission, which underscores the importance of considering dopaminergic pathways in managing apathy in older adults.

